# An Institutional System Proposal for Advanced Occupational Safety and Labor Standards in the Turkish Construction Industry

**DOI:** 10.3390/ijerph192215113

**Published:** 2022-11-16

**Authors:** Nihan Yıldırım, Derya Gultekin, Doğan Tilkici, Dilek Ay

**Affiliations:** Department of Management Engineering, Faculty of Management, Istanbul Technical University, Istanbul 34367, Turkey

**Keywords:** construction, Turkish construction, institutional system, labor standards, occupational health and safety policy, decent work

## Abstract

The Turkish construction industry is problematic with its inferior occupational safety practices and labor standards. This paper explores the current institutional system’s problems and designs a national institutional system to improve labor conditions in the Turkish construction industry. The study applies cause-and-effect analysis, stakeholder analysis, and information flow analysis based on the thematic literature and regulation reviews and the data collected from expert interviews. Findings revealed that the industry represents a drastically problematic context with high rates of occupational accidents, job insecurity, and excessive working time, and the inspection and enforcement system is still immature, calling for additional institutional arrangements to establish a collaborative and sustainable environment. There is a need for a holistic, multi-dimensional, and systematic perspective to develop coordination and inspection mechanisms in the sector. The paper proposes an institution and scorecard design by applying a quality function deployment framework matching needs and systemic functions that can overcome the existing deficiency in labor conditions. The paper contributes to filling the gap in the literature on the multi-dimensional, systematic institutional perspective to develop coordination and inspection mechanisms in the construction industry and proposes an institutional system example that could be adapted to other national contexts.

## 1. Introduction

Work conditions have worsened globally in the construction industry since the 1970s due to the changes in employment regimes in terms of wage schemes, work hours, social security inclusion, and occupational health and safety (OHS) [1,2]. The rapidly disseminated subcontracting led to the recruitment of workers with lower-tier subcontractors who do not have the competency to adapt to labor standards and intend to dismiss them when not appropriately inspected [3]. Despite its extensive employment and value-added contributions [4,5], the construction industry attracted attention negatively as a leader in occupational accidents [6]. The compelling problem of high rates of accidents is expected to be aggravated by global recessions and urgently calls for a solution [2,7].

The Turkish construction sector was a so-called highly functioning engine that outputted high economic growth rates in the post-2000 period. Before the economic slowdown that started in 2017, its shares in the GDP and employment reached 9.6% and 7%, respectively [8]. As of January 2022, 1,400,000 “registered” construction workers were employed in Turkey [9]. The Turkish construction industry holds top ranks globally in the total and fatal occupational accident rates and indecent work and employment conditions [10,11,12].

In the Turkish construction sector, subcontracting shapes the employment structure; unregistered and insecure working is ordinary and unionization is rare, bearing a low bargaining power [13,14,15,16]. The industry’s labor standards’ deficiency undermines its sustainable development performance [17]. Given the job and social insecurity and the workforce’s low education, the sector becomes trapped in a vicious cycle of low skill–low productivity, failing to initiate a sustainable growth pattern based on high value-added production. Hence, the industry needs a more secure and safer work environment that fulfills the “decent work” conditions defined by the ILO [18] to achieve the specific targets of the new international program of sustainable development [19]. Additionally, low labor standards in the construction industry are a matter of non-conformance to the United Nations (UN) Sustainable Development Goal (SDG) no. 8 “Decent Work and Sustainable Growth”, which focuses on promoting inclusive and sustainable economic growth, full and productive employment, and decent work for all [20].

Previous research on Turkey’s construction industry’s work standards mainly focuses on one particular dimension of issues by utilizing a single-discipline approach. Therefore, there is a need for holistic, multi-dimensional, and systematic studies to develop efficiency, coordination, and inspection mechanisms in the sector. It is essential to examine the stakeholders, relationship networks, and regulatory framework as an integrated national institutional system that determines work standards in an industrial context. This study addresses problems related to occupational safety and labor standards by searching for their roots in the current national institutional system to improve them in line with international standards. To this end, it develops a system proposal to systematically address and solve the problems of inferior labor conditions in the Turkish construction industry, using a system development approach. To the best of our knowledge, this study is unique for providing a case using system analysis and design methods to explore the problems and propose institutional system improvements to raise labor standards in the sector. We firstly collected primary data through in-depth structured interviews with stakeholders. Secondly, we conducted an institutional system analysis on the regulations and stakeholders to shed light on the current institutional system. Additionally, flow chart modeling elaborated the directional interactions between stakeholders; the cause-and-effect analysis revealed the root causes of chronic labor standard problems in the industry. Lastly, based on the findings of the current system analysis, design requirements are defined and solution proposals are designed by the quality function deployment method. This paper’s original contribution is that it focuses on the overall national institutional structure of occupational safety and labor standards in the construction industry and includes simultaneously the stakeholders’ views for addressing problems and proposing systematic solutions.

The article reflects the institutional needs targeted by the ILO, referring to the UN’s SDG no. 16 “Peace, Justice and Strong Institutions” [20]. The ILO [21] listed the “C144 Tripartite Consultation (International Labor Standards) Convention, 1976” and “R205 Employment and Decent Work for Peace and Resilience Recommendation, 2017” as instruments relevant to Goal no. 16, aiming for institutional improvements in labor standards. Accordingly, the proposed institutional system provides the needed inputs to policymaking and road-mapping processes. The system approach that we utilized in the case of the Turkish construction industry can also be adapted to other national/regional contexts. The study used the evidence from the Turkish case to show that multi-layered labor problems faced in the global construction industry could be addressed via a holistic institutional system approach.

### Literature Review: Labor Standards in the Construction Sector

Subcontracting has become a permanent employment strategy in most countries due to the peculiar nature of the construction product which is immobile, geographically dispersed, and varies in size and composition [22]. The requirements of particular expertise, advanced equipment, and enormous investment cost have made multi-layered subcontracting a norm in the industry [3]. Workers are employed in insecure conditions in the subcontracting system far from collective bargaining opportunities, and they can limitedly benefit from OHS training. Poorer OHS is a critical consequence of subcontracting. The “payment-by-results system” pushes subcontractors to work excessive hours, and intense competition drives down the price of subcontractors’ services, thus reducing the priority placed upon OHS [23,24].

Multi-layer chain subcontracting means multiple subcontractors from multiple companies simultaneously work on the same site [25]. Multi-layered subcontracting challenges the project management, coordination, and supervision in construction for all partners (contractors, clients, and project management teams). It blurs wage and compensation claims [3] and increases OHS incidences and accidents, especially for subcontractors’ employees [25]. Thus, by the diffusion of subcontracting, the construction industry becomes characterized by an increased risk of work accidents and worker rights violations [26,27,28]. The adverse OHS effects of subcontracting have been noted in various country studies such as in Spain, China, Australia, Hong Kong, the United Kingdom (UK), and the United States of America (USA) [29,30,31,32,33,34,35].

The construction industry also heavily relies on international and in-country migrant workers that are more prone to lower work standards and accidents [36,37,38,39,40,41]. Migrant workers, with their low qualifications and limited job opportunities, become available for informal employment, which offers low cost and responsibility for the employers but bears high fatal risks for the workers [42,43].

Furthermore, even if there are sufficient legal regulations, construction workers are still forced to work under unsafe and insecure conditions in many countries. As the necessary site inspections are not carried out, and no penalties are imposed, the legal minimum wage, weekly working hours, overtime pay, paid vacation leave, and social security rights are violated [1,44]. Even in the EU27, the construction sector is the leader in fatal occupational accidents (one-fifth of 3332 fatal accidents in 2018 while Germany and the Netherlands recorded the lowest rates with no more than one fatal accident per 100,000 employees) [45]. The Turkish construction sector performs extremely poorly in the fatal work accident rate. Although the sector constituted 7% of the workforce in 2018, it is distressing that it reached an enormous rate of 38.4% in total fatalities [46], which shows that the construction sector is the worst-performing and a victimizing industry in the Turkish economy. Moreover, current occupational accident statistics are far from reflecting the true extent of the problem in the sector where unregistered and migrant worker employment is pervasive [13,15,16,47]. In addition, the sector has higher weekly work hours than the EU, and the legal work hour limits are exceeded in practice, calling for an urgent improvement in the inspection and enforcement system.

Bosch and Philips [4] present an in-depth comparison of nine countries’ construction sectors (the Netherlands, Germany, Denmark, Canada, Australia, Spain, the USA, the UK, and South Korea). They conclude that countries might take one of the two growth paths: “low” or “high” roads in labor standards. The low path refers to an industry characterized by poorly educated and skilled workers with low wages but higher numbers of workers. However, the high road represents an industry with highly skilled workers with higher costs and wages but more output [4]. Labor standards in Turkish construction can be classified under the low-road strategy with low conformance to labor standards, bearing many adverse facets. Subcontracting has shaped an intensified informal, insecure, and ununionized employment regime. Working for a subcontractor significantly limits the workers’ employment and organizing rights [48]. The education status of Turkish construction workers is also relatively poor: 72% had less education than high school (2017), while vocational high school education is needed for a skilled workforce. Additionally, only 68% of the employees work as registered [49]. The industry normalized inappropriate on-site accommodation conditions, long working hours, and employment of immigrant workers, causing higher risks of occupational accidents [50], similar to the conclusions of Harroff-Tavel and Nasri [51]. The employment of an increased number of immigrant workers further deteriorates conditions of wages, working time, job security, and social security [52,53]. With the Syrian immigration, competition among construction workers intensified, and local workers’ bargaining power decreased [50]. All those adverse circumstances reduce production quality (causing a low-productivity/low-quality helix) and undermine the institutionalization and professionalism in the sector [54].

The negligence of basic workers’ rights in Turkish construction impels various researchers to study this topic. Given that it is the deadliest sector, most articles take work safety as their primary concern by exploring the dimensions of the nature of construction, education, subcontracting, and the time, place, type, and causes of workplace accidents. Almost all reasons for low labor standards point to other problems, such as the organizational structure and working culture [55], and the lack of unionization that limits the influence of the worker unions and undermines the collaborative workplace. Duman and Etiler [56] recommend a better system to keep track of accidents by workplace medical professionals that are equipped with government responsibilities. Studying the Work Safety Act, Baradan et al. [57] emphasize that a regulation system specifying technical details of precautions is lacking despite the government’s responsibility to employ and force work safety regulations. Akbıyıklı and Dikmen report that the construction managers are unaware of their responsibilities, and lack knowledge of OHS and labor standards regulations [58]. Yılmaz [59] stresses that the inspection system is insufficient to force companies to obey the work safety regulations since the inspections are not planned in tune with the sector’s size, risk levels, and individual companies. Execution of penalties is not working correctly, either [60]. In addition, OHS specialists’ role and effectiveness in preventing OHS problems remain insufficient as most OHS specialists have limited competency, knowledge, and experience, accompanied by a lack of autonomy due to their work contracts with employers [61]. Compared with the USA, the Turkish construction sector is far from employing a compensation insurance system, conducting effective inspections and enforcement, and ensuring all stakeholders’ awareness of work safety problems and possible solutions [62]. Hence, a dynamic insurance system setting the worker’s insurance premium by the accident rate in the workplace is strongly needed [63].

In brief, previous research on labor standards in the sector primarily focused on specific problems such as high occupational incidents and long working hours, subcontracting, lack of unionization, lack of individual employment rights, and employment of immigrant workers. Some other studies explored the causality between those problems and the legal and institutional systems and concluded that there are some insufficiencies in the regulation system, inspection system, and execution of penalties [57,59]. The studies also revealed that the existing OHS problems are linked to the fast growth of the industry, workforce structure, and low performance of control mechanisms [47,64]. For the Turkish construction industry, there is a significant need for an institutional system that can provide legal status to OHS specialists and inspectors, ensuring their autonomy and authorization for inspection reporting and reinforcement while providing monitoring and inspection of their activities by public authorities [61]. Additionally, all stakeholders of construction projects should be aligned and collaborate within the same systematic framework for holistic OHS management [58]. The ILO [2] lists recommendations for solving OHS issues in the global construction industry and underlines that sector stakeholders should work in cooperation with inspectors to enhance safe and healthy environments.

The above-mentioned highlights from the previous theoretical background refer to a solicited need for institutional system analysis and proposed system design. However, no previous study methodologically analyzed current conditions and possible improvements from an institutional and legal perspective, using basic system development methodology. This study aims to develop a national institutional system to enhance labor conditions in the Turkish construction sector while holistically considering all stakeholders and updating labor legislation by proposing an institution and scorecard application design. The key research questions are the following: (1) What are the problems and requirements of the current institutional and regulative systems which govern the OHS and labor standards in the Turkish construction industry? (2) Which solutions can we propose to improve the current system to meet global labor standards set by the ILO? (3) Can traditional engineering system development methods be applied to an institutional system design for solving OHS and labor standards problems?

## 2. Materials and Methods

Secondary data were derived from the review of the literature and official reports of institutions (e.g., the ILO, TUIK, and the Republic of Turkey Ministry of Labor and Social Security), as referred to in the literature review section. Primary data were collected from sector shareholders through in-depth structured interviews, referring to the qualitative research interview methodology of Kvale [65]. The interview questionnaire was built upon the topics and dimensions derived from the literature review’s content analysis. The in-depth interviews were conducted in Istanbul between 2019 and 2020 with seven highly experienced representatives and experts on OHS and labor standards in the construction industry of Turkey.

By exploring the labor standards problems and their solutions in the Turkish construction industry, this study adopted a socio-technical and interdisciplinary approach by applying a thematic literature analysis, regulative analysis, semi-structured interviews for data collection, and analysis by thematic coding and triangulation of the findings from these data sources. Stakeholder analysis and cause-and-effect analysis are applied to structuring the findings. System design methods such as quality function deployment (QFD) and use case diagrams are used for explaining the requirements analysis and matching requirements with the proposed system solution components. A QFD intervention matrix is also utilized for the validation of the proposed model for its conformance to needs, referring to the System Engineering Body of Knowledge [66]. However, the actual system validation requires demonstrating that the product, service, or enterprise satisfies its system; stakeholder requirements verification of such a system is only possible after the actual implementation of this system in real life, which is beyond the scope and capabilities of a logical system design study.

**Figure 1:**
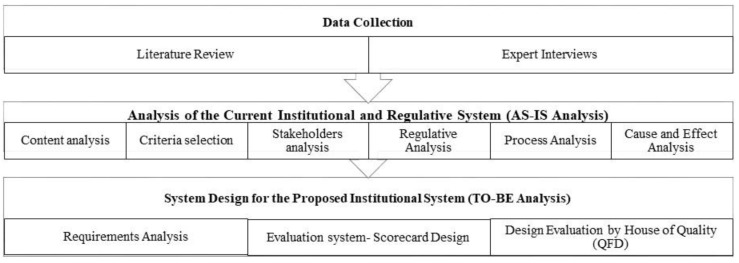
Methodology of the research.

Figure 1 presents the methodological steps involved in the study. Current state (AS-IS) analysis includes the analyses of stakeholders, regulations, processes, and causes-and-effects based on the inductive findings from the content analysis of the literature and expert interviews. Stakeholder analysis is used to understand key actors whose interests must be considered during policy development to gather and analyze qualitative information [67]. It centers on a mean or group of means to obtain information about particular figures (people or a group of people) to learn their motivations, actions, benefits, or relations with each other. It is also used to evaluate the impact of stakeholders to be referred to in decision-making and implementation processes on systematic change [68]. The regulative analysis of labor standards assesses current regulations in comparison to the ILO and EU directives. Process analysis expresses the direction of activities, processes, their order, and fundamental connections as a graphical demonstration method that is simple and clear [69]. Process flowcharts are used to analyze the information and workflow in the current institutional system for labor standards. The cause-and-effect diagram employs graphical illustration to demonstrate relationships by focusing on causes; it is used when displaying the effects, factors, or causes of a specific problem [70]. The technique is used backward from the situation to the root cause while uncovering every cause leading to the problem [71]. In the future state (TO-BE) analysis, the requirements of the institutional system are identified based on the results of the AS-IS analysis. As an essential tool of the QFD method, the house of quality matrix evaluates the new system in terms of design/customer requirements, relative importance, the correlation between the new system’s parts, and benchmarks with the old system [72].

After the selection of criteria, the interviewees (presented in Table 1) participated in the semi-structured interviews and the selected five criteria (which are explained in Section 3.1.1) were used for the questionnaire. The interviewees also provided data for the stakeholder analysis by rating stakeholders’ influences by their authority and enforcement roles in governing and practice on a linguistic scale between low, medium, and high. The study’s major limitation in data collection is that the employers’ representatives could not be interviewed despite researchers’ efforts and invitations to the employers’ union.

Four researchers independently coded the findings from the interviews under five criteria and then by the themes of problems and causes of failures derived from the literature and regulative content analyses for the current institutional system for labor standards in the Turkish construction industry.

## 3. Results

### 3.1. AS-IS Analysis of OHS and Labor Standards in the Current Institutional System: Inductive Findings from the Content Analysis

#### 3.1.1. Labor Standards Criteria Selection, Data Collection, and Analysis

From a complete list of labor standards determined by the ILO [73], we selected the criteria set to be used in analysis and design. The ILO’s list includes the following: Freedom of association, collective bargaining, and industrial relations; Forced labor; Elimination of child labor and protection of children and young persons; Equality of opportunity and treatment; Tripartite consultation; Labor administration and inspection; Employment policy and promotion; Vocational guidance and training; Employment security; Wages; Working time; Occupational safety and health; Social security; Maternity protection; Social policy; Migrant workers; HIV and AIDS; Seafarers; Fishers; Dockworkers, Indigenous and tribal peoples; Specific categories of workers. The complete list of subjects was discussed with the construction and OHS expert, law expert, and academician, and they rated the criteria on a scale of 1—crucial, 2—moderate, and 3—not critical for their importance in balancing the high productivity for growth and workers’ mental and physical health protection in the industry. The selected final criteria set includes the following standards:

(1) Working and Rest Time: Limiting working hours and protecting rest time constitute critical aspects of creating better labor conditions [19].
(2) Job Security and Registration to Social Security Institutions: The aspects of job security are registered employment and the ease of individual and collective dismissals. According to the OECD [74], employment protection legislation (EPL) is an indicator of job security. International and national legislations are in place to restrain companies from making arbitrary dismissals [75].
(3) OHS: It is considered the most critical and immediate criterion for decent work conditions [19]. Especially in the construction sector, where jobs are hazardous, leading to a high fatality rate in occupational accidents in unprepared workplaces, OHS is a primary indicator of workplace standards.
(4) Education and Vocational Training: Vocational training and education prepare individuals for the profession through formal or non-formal education and improve their professional capabilities [76]. Vocational education provides the necessary knowledge, skills, attitudes, and values to individuals for developing physical, social, cultural, and economic capabilities, enabling their adaptation to new occupations. Human Resources Development Convention 1975 (no. 142) of the ILO [73] also requires ratifying nation states to develop policies, programs, and complementary systems of vocational guidance, training, and education for workers.
(5) Employee Services: Also called worker on-site accommodations, these are the sets of practices to provide the worker with safe and sufficient working conditions outside of active working hours [77]. These practices may include but are not limited to access to fresh water, break rooms, locker rooms, adequate housing if needed, toilets, showers, and hot water. The existence, accessibility, and quality of those services are essential. Employee services can be associated with the hygiene theory of Herzberg [78], suggesting that some fundamental factors (hygiene factors) cause detrimental effects when they are not fulfilled while not having additional positive impacts when they are fulfilled. Despite being excluded in the ILO Convention no. 122 [73], employee services are included as a criterion in this study, for the accommodation service problem is still evident in the Turkish construction industry [50].

#### 3.1.2. Stakeholder Analysis by Roles and Responsibilities

Stakeholder analysis is imperative to comprehend occupational accidents since the lack of coordination between main actors and institutions of the industry is among the key reasons. The intended system design proposal should also offer a well-functioning integrated system considering the importance of cooperation between stakeholders. Accordingly, based on the content analysis of the literature, reports, regulations, and opinions of interviewed experts, an analysis of stakeholders identified the roles and responsibilities of actors and their degree of influence on labor standards (Table 2). The interviewed experts rated the stakeholders’ influences by their authority and enforcement roles in governing and practice on a linguistic scale between low, medium, and high. Table 2 shows shared roles and responsibilities in gray areas.

As Table 2 presents, the Institute of Occupational Health and Safety is responsible for examining OHS in workplaces and preparing projects for ensuring OHS. On the other hand, OHS specialists audit and report OHS practices in workplaces. The institute and specialists have similar roles but different coverage and responsibilities. On the other hand, the Work Inspection Institute examines labor standard practices, having a similar role to the Institute of Occupational Health and Safety. However, the coverage of the Work Inspection Institute is based on work hours and job security. Hence, labor standards in workplaces are inspected by the inspectors of both institutions, but in different procedures and scopes. There are two educational bodies in the institutional system. The Occupational Qualification Institution act as the certification body for vocational education. The Work and Social Security Training and Research Centre also provides training and information dissemination; however, it focuses on working life and social security. Similar to this center, the Social Security Institute has the role of increasing awareness of social security rights. Labor unions, the ILO, and NGOs are also working on awareness raising and information dissemination on OHS and labor standards. For policy-making, the Social Policies Board focuses on policy recommendations for low-income populations. The Ministry of Labor and Social Services is the regulatory body of the institutional system, but the Presidency of the Republic of Turkey directs the labor standard-related issues to this ministry. As can be traced from these highlights in Table 2, most stakeholders perform unique roles in the system. However, in the above-mentioned instances, some roles overlap, though the actors’ responsibility levels differ.

In Turkey, the Ministry of Family, Labor, and Social Services has the executive power for providing labor standards. The Labor Inspection Board and Social Security Institute establish and maintain the inspection and/or enforcement system. The board is responsible for working hours and OHS criteria. It employs labor inspectors who perform workplace audits, being equipped with power tools such as the right to inspect workplaces anytime and close operations if necessary. Governors perform the enforcement resulting in operation shutdown decisions. However, interviews with work inspectors revealed that the governors sometimes do not carry out the instruction, aligning with the fact that in 2016, 46 of 467 cease requests by work inspectors for construction firms were not executed by 36 different governors in Turkey [60].

Previous literature provided insights into the performance indicators of the work inspection and enforcement system of Turkey. A minimum rate of 20,000 active workers per inspector should be achieved for an adequate inspection system in developing countries [79]. However, this rate was 24,059 active workers per inspector in Turkey (2013) [59]. In 2016, only 96,359 employees were inspected in the Turkish construction industry [60], constituting a mere 0.48% of total workers. The insufficient number of work inspectors and inspections acts as a barrier to the inspection and enforcement system.

The Social Security Institute intermediates job security by making dismissals difficult and inspecting the workers’ social security registration status. Social security inspections take place in workplaces and construction sites. When unregistered workers are detected, the institute charges a fine to the employer. In 2017, only 67.5% of the construction employees were registered for social security [49], indicating low quality, fines, and/or frequency of inspections. Therefore, the inspection and enforcement system is inadequate to provide proper labor standards.

#### 3.1.3. Regulative Analysis of Labor and OHS Regulations in the Construction Industry

Turkey’s labor legislation is benchmarked with the ILO conventions and EU regulations for each criterion to determine whether there are any discrepancies requiring new legislation in Turkey. The comparative regulative analysis findings are below:

Working Time and Rest: In Turkey’s Labor Act [80], the weekly maximum working time is 45 h, which is fair but still behind (5 h more than) the ILO standard (C047—Forty-Hour Week Convention, 1935) [81]. On the other hand, working time in Turkey is 3 h lower than the EU (Directive 2003/88/EC of the European Parliament and of the Council of 4 November 2003 concerning certain aspects of the organization of working time, 2003) [82]. Vacation days can be defined as the minimum days or working weeks of paid vacation. In terms of annual vacation days, Turkey is considerably worse with just 14 days [80] compared to the ILO’s 3 working weeks (Holidays with Pay Convention, 1970) [83] and the EU’s (Directive 2003/88/EC) 4 working weeks [82].

Job Security: EPL measures the procedures and costs involved in dismissing individuals or groups of workers and the procedures involved in hiring workers on fixed-term or temporary (precarious) work agency contracts [84]. By EPL points, we can compare countries regarding employees’ job security. The scale for restrictions against worker dismissals is between 0 (the lowest) and 6 (the highest). Turkey’s score is 2.33.

OHS: Turkey has adequate coverage in terms of OHS and works safety [85] when compared with the ILO’s conventions (no. 155—Occupational Safety and Health Convention, 1981 and no. 161—Occupational Health Services Convention, 1985) [73] and the EU’s Directive (no. 89/391/EEC) [86].

Education: Vocational education is compulsory in every case under consideration. According to the Vocational Training Act of Turkey [87], vocational education requires 12 years following the duration requirements of the OECD [84].

Employee Services: The ILO [77] recommends accommodating workers assigned to another country or city. In Turkey, the Work Health and Safety Act [85] and related regulations require an employer to provide hygienic and safe accommodation conditions for the workers.

#### 3.1.4. Information and Work Flows in Current Institutional System for Labor Standards and OHS Inspection and Enforcement Systems

**Figure 2:**
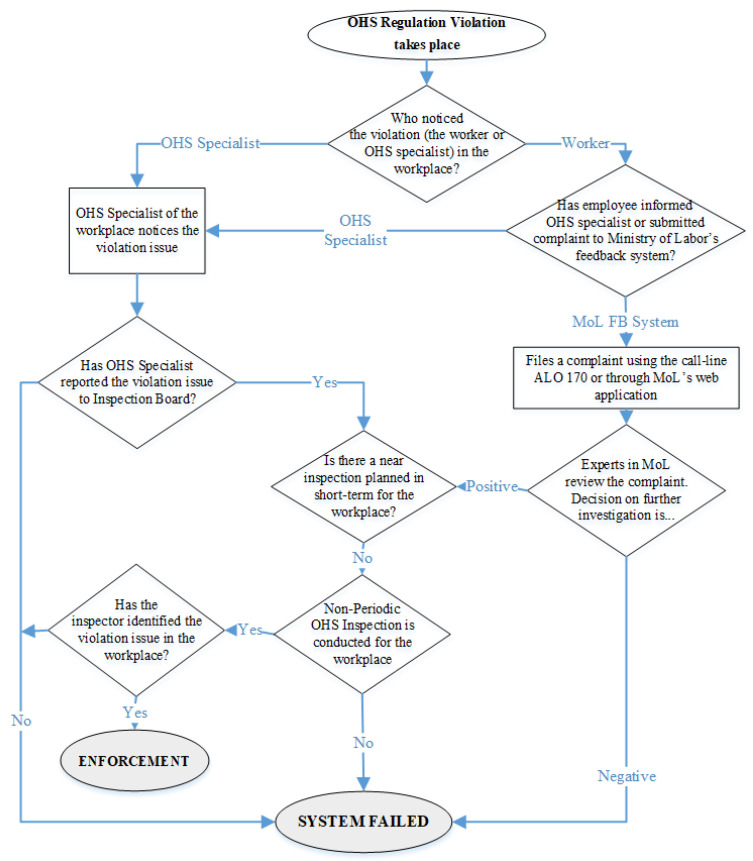
Process Flowchart of Institutional System for OHS Regulation Violations.

**Figure 3:**
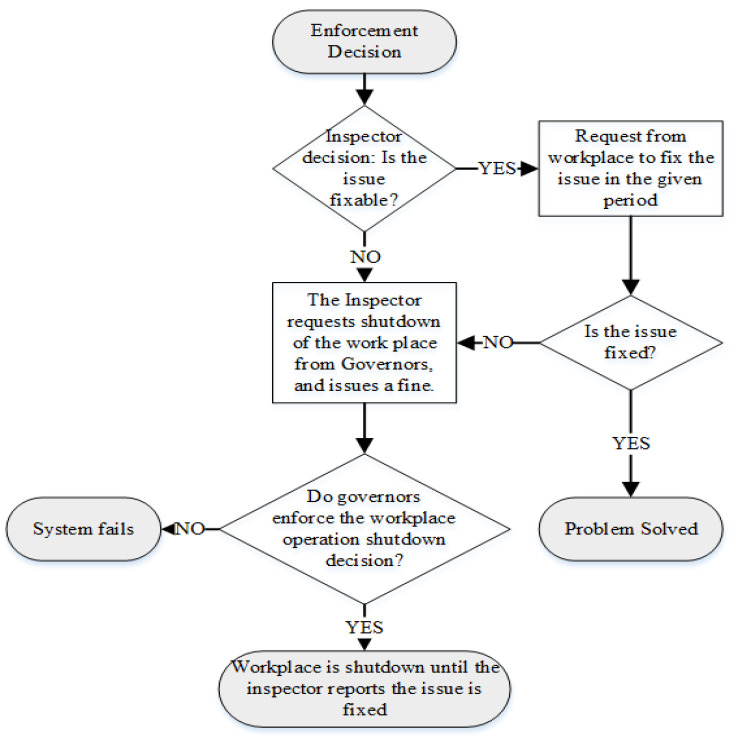
Flowchart of the Post-Enforcement Decision Process in the OHS Institutional System.

**Figure 4:**
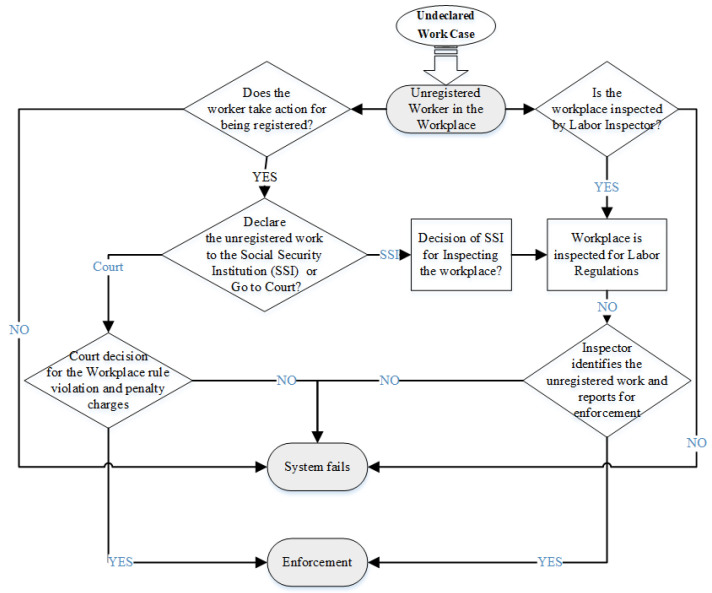
Flowchart of the Unregistered Workers Case Process in the OHS Institutional System.

Based on the findings from the stakeholder analysis, regulative analysis, and expert interviews, information and workflow of the current labor standard practices in the selected four criteria are constructed (Figure 2, Figure 3 and Figure 4). These charts show how non-compliances are processed and how the violations and errors are stopped or unnoticed in the Turkish construction industry’s labor and OHS institutional system. Colored boxes indicate system failures in the implementation of OHS regulations. The failure conditions occurred as the lack of inspection; the violation complaints remaining unreviewed by the authorized experts; undetected problems by the inspectors; and the cases in which the governors do not take enforcement action.

As Figure 2 displays, the current institutional system for OHS regulation violations in Turkey has four main causes of failure in the process flow. The first failure cause is the adverse attitude of workplace OHS specialists towards reporting the violation issues to the Inspection Board. This can be due to many reasons: (1) As revealed in the interviews with OHS specialists and discussed in previous literature, OHS specialists are given limited power due to their minimal authority and entitlement challenges [59,61,88,89]. (2) OHS specialists’ role and effectiveness in preventing OHS problems remain insufficient as most of them have limited competency, knowledge, and experience, accompanied by a lack of autonomy due to their work contracts with employers [61]. Secondly, whether reported by the employee or by the workplace OHS specialist, the violation of an OHS regulation should result in an inspection. If there is not a planned inspection in the short term, then the Inspection Board should assign OHS inspectors for a non-periodic inspection. However, in some instances, these inspections do not take place, leading to a system failure. Thirdly, even if a non-periodic inspection takes place following the violation claim, the assigned OHS inspectors may not identify and report the issue due to their lack of knowledge and work intensity. As explained in Section 3.1.2, Turkey, as a developing country, hardly complies with the ILO’s [79] principle on the minimum rate of 20,000 active workers per inspector for an adequate inspection system [59]. Fourthly, experts evaluating the complaints coming from the Ministry of Labor’s feedback system might overlook the claims and they might not enforce an inspection for the issue. The low rate of inspection in Turkey is a fact [60], creating an adverse attitude in institutions for requesting more inspections.

For the post-enforcement decision processes (Figure 3), as the predecessor of system failure, governors might refuse the inspectors’ requests for operation shutdown of the workplaces whose OHS regulation violations are identified. The underlying economic, political, and cultural factors and possible biased decisions of governors about some employers could cause this failure.

Figure 4 explains the process regarding unregistered workers in the OHS institutional system of the Turkish construction industry. In this process, one predecessor of system failure is the lack of court decisions for rule violations and penalty charges. Here, the justice system and independency of labor courts are worth exploring through in-depth studies. On the other hand, the lack of labor inspection in workplaces also makes the violations invisible and deepens the unregistered employment in the sector. Additionally, similar to the findings from Figure 2 on OHS inspectors, the quantitative and qualitative insufficiencies of inspectors stand as a barrier to the prevention of unregistered employment.

#### 3.1.5. Cause-and-Effect Analysis

The cause-and-effect analysis is based on the literature content analysis, regulative analysis, and interview findings. Twelve causes of multi-layered problems are derived from the current institutional system in Turkey’s construction industry. During the analysis of the interview data, four researchers coded the statements from the interviews under these causes. The content analysis and interview coding results are given in Table 3, showing the matching themes from the literature review, regulative analysis, and interview coding by researchers. The interviewee’s concordance is also shown in the table. The last column shows the ratio of researchers who had coded the interview statements under the mentioned theme, representing the concordance of the ratings. In this way, three different sources of information are triangulated by comparative analysis and cross-checking the findings across themes under the five criteria of labor standards in the construction industry.

As Table 3 displays, all causes derived from the literature content analysis and interview coding are aligned with each other, while some new themes emerged from the interviews, not reported in previous literature. However, for some themes under OHS criteria, Employee Services and Education and Vocational Training, the regulations in Turkey are not adverse, but the literature analysis and interviews pointed out insufficiency in practice.

**Figure 5:**
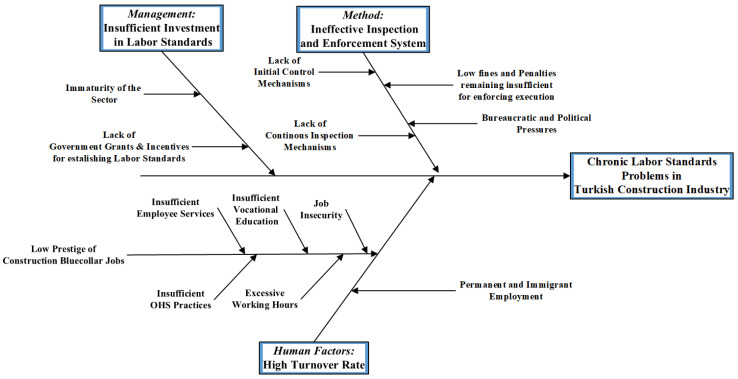
Cause-and-Effect Diagram.

Based on the analyses of the literature, stakeholders, regulations, and interviews, the cause-and-effect analysis explains the linkages between the multi-layered problems and their causes in the current institutional system in Turkey’s construction industry. In this study, the man, machine, material, method (4M) framework on cause–effect analysis is used, which is well known for quality control and analysis of the causes of accidents [90]. The 4M framework also provides a useful perspective for identifying wastes and Kaizen points in a system [91]. We found three primary causes of chronic low labor standards: An ineffective inspection and enforcement system (method); sector-wide high turnover rates (human factors (formerly called “man” but revised by authors following diversity principles); and insufficient investment in labor standards (management) (Figure 5). The causes regarding material were not diagnosed by the analysis, hence the fourth M is not applied in Figure 5.

The primary causes and subcauses of chronic labor standard problems identified by the cause-and-effect analysis are elaborated below:

1. An Ineffective Inspection and Enforcement System: Regulative innovations will not be successful in practice if they are not supported by inspection and enforcement exercises [84]. Inspection systems must adequately recognize labor standard problems. The contrast between adequate regulations and inappropriate practices noted in the previous sections indicates that the ineffective inspection and enforcement system causes low labor standards in the sector. This problem’s root causes are classified as follows: a lack of initial control mechanisms; bureaucratic and political barriers; deficiency of a continuous inspection system; and low fines.
  - Lack of Pre-Operational Check Mechanisms: As stated by the interviewed labor inspector, there is no pre-operational compliance check mechanism in the current inspection and enforcement system. Due to the low inspection rate addressed in both interviews and the literature review, this issue becomes even more severe by bearing the high risk of unnoticed breaches in the regulations. Although prevention should be a primary goal [59], the lack of an initial control system as a preventive measure constitutes a significant deficiency in the inspection and enforcement system.
  - Bureaucratic and Political Barriers: The interviewed experts underlined that political power holders hinder the inspections’ conduction under various employer groups’ influences, as also claimed by Gürcanlı [92]. Independent from the executive power, political parties, pressure groups, or other influencers, the system should enable objective and unbiased inspections.
  - Lack of Continuous Inspection Mechanisms: The sector’s current inspection system has non-continuous monitoring, referring to control applications at intervals rather than throughout all operation processes. Considering that the interval inspection frequency is low, continuous monitoring mechanisms should be established to raise labor standards through inspection.
  - Low Fines: A frequently expressed problem in the interviews is that legal penalties for labor practices are cheaper than establishing labor protection mechanisms in workplaces. Thus, fines, which act as negative reinforcement, are not effective in forcing employers to practice the regulations in Turkey’s construction firms.
2. Sector-Wide High Turnover Rates: Most of the workers (around 70%) [13,48] employed in the Turkish construction sector are seasonal workers who return to their hometowns after the completion of the project. Expert interviews also support pervasive seasonal and project-based working in the industry that results in a high turnover rate and causes low productivity, confirming Guthrie’s discussion [93]. The interviews revealed that the high turnover rate is caused by the fact that workers perceive their work “as a source of income to meet their necessities in the short term” rather than a profession. Due to this perception, workers are unmotivated to become trained and advance their vocational skills and knowledge, resulting in OHS risks due to a lack of expertise.
  - Low Prestige of Construction Jobs: “Low-prestige job” perception of construction work is an intermediate problem leading to high turnover rates in Turkey’s construction industry. The lack of hygiene motivation factors of job security, working hours, employee services, and OHS causes job dissatisfaction, while other motivation factors such as achievement, recognition, and career advancement lead to job satisfaction [78]. Vocational education and training establish achievement, recognition, and progress [94]. The root causes of workers’ common dissatisfaction with the profession in Turkey’s construction sector can be listed as primitive employee services, excessive work hours, low job security, poor OHS, and insufficient vocational education.
3. Insufficient Investment in Labor Standards:
  - Immature Sector: While the construction sector highly contributes to GDP in the initial phases of economic development, its contribution decreases after a particular threshold is achieved [95]. Turkey’s construction sector has not reached maturity yet and records higher growth rates than that of mature economies [96]. The industry has offered accessible entry opportunities to investors during the high growth trends in the 1980s and post-2001 periods, allowing them to practice low labor standards to sustain their competitiveness [48]. The expert interviews showed that most Turkish construction firms seized those growth periods’ short-term business opportunities and did not prioritize improving labor standards since it required investment and long-term orientation.
  - Lack of Governmental Grants: Small-to-medium companies without sufficient resources constitute a large portion of Turkey’s construction sector [97]. For improving labor standards, most companies are dependent on governmental grants that have remained insufficient.

### 3.2. A Proposed Institutional System Design for Improving Occupational Safety and Labor Standards: To-Be System

After determining the root causes of the problems, identifying system requirements for the removal of these causes is the next step towards proposing an institutional system change that might solve chronic labor standard problems in the Turkish construction sector.

#### 3.2.1. Identification of Requirements for Redesigning the Institutional System

Table 4 presents the design requirements corresponding to the root causes addressed in Figure 5. We excluded the root cause of the “immature sector” since this is a structural factor requiring long-term improvements beyond the institutional system advancement boundaries. Responding to the deficiencies in the inspection and enforcement system, some design specifications on effective pre-occupational control mechanisms and objectivity measures are proposed. Fairness of fines and effectiveness of inspection systems are also required in this domain. On the other hand, the high turnover rate was found as the main cause in Figure 5, which can be overcome by adequate working hours, employee services, OHS measures, vocational education, and job assurance. Despite the Work Health and Safety Act [85] and relevant regulations in Turkey that stipulate the provision of hygienic and safe accommodation conditions, expert interviews revealed that the accommodation problem of construction workers is still prevalent, especially in micro and small-scale firms. Poor accommodation conditions can be improved through an adequate inspection and enforcement system. As a structural problem of the industry, insufficient investment in labor standards is also addressed in the cause-and-effect analysis. In response to this issue, governmental grants can be offered, though the state intervention might remain insufficient and an actual solution to this issue can only be provided by the competitiveness and efficiency improvements of the industry in the long term, which goes beyond the institutional design component.

#### 3.2.2. Evaluation of the Requirements and Building the Design Components of the Proposed System

**Figure 6:**
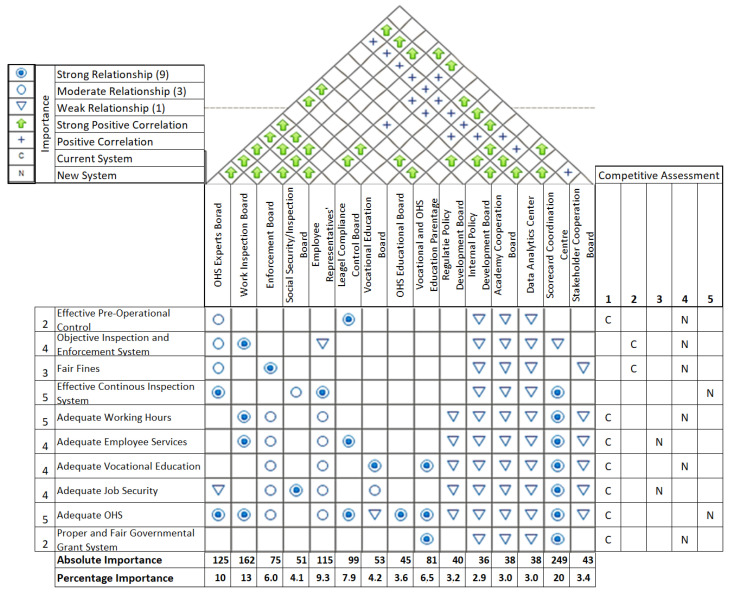
Quality Function Deployment Canvas (House of Quality).

A house of quality application as a derived version of QFD (Figure 6) conceptualizes the institutional system design components. The left block of this house consists of design requirements and their importance. The quality house’s central block demonstrates relationships between the design requirements and components in a matrix form. For example, the current system is scored as 1 (very weak) for meeting the “Effectiveness of Continuous Inspection System” requirement. In contrast, the proposed new system is scored as 5 (very strong) for meeting this requirement. The interviewed experts then assigned the relative importance of each requirement on a 1–5 scale (1—“too low”, 5—“very high”). The first column of Figure 6 shows the importance of the design requirements by the arithmetic averages of the expert ratings. The lower block measures each design component’s importance.

In the house of quality, the roof represents a correlation matrix showing the relations between the designed components in the upper block (which are the organizational units proposed in our solution). The correlation matrix shows a positive correlation between the Coordinated Inspection and Enforcement Committee and others, pointing out their cooperation’s importance. The scorecard application center strongly correlates with various units, reflecting the importance of inter-institutional integration. The lower block measures each design component’s (proposed organization units’) importance. The quality house’s main and lower body indicates that the scorecard application nearly achieves 20% importance, making it crucial to the institutional system. Hence, the critical design components with high priority are scorecard application center, labor inspection board, and OHS experts board. The right block of the quality house displays the competitive assessment of the new (N) and current (C) systems by their ability to meet the requirements on a Likert Scale (1—very weak to 5—very strong). The competitive assessment indicates that the new system surpasses the current system in all criteria.

#### 3.2.3. Validation of the Proposed Solution

According to the System Engineering Body of Knowledge [66], system validation requires demonstrating that the product, service, or enterprise satisfies its system and stakeholder requirements. Each requirement derived from the stakeholder analysis and cause-and-effect analysis was matched to a solution component in Figure 6. Hence, we can assure that the proposed system “does the right task”, validating the proposed solutions and functions. QFD and similar design methods enable visualizing and cross-checking function requirements in the same framework for logical validation.

#### 3.2.4. Design of the Coordination Organization: Integrated Labor Standards Institute

**Figure 7:**
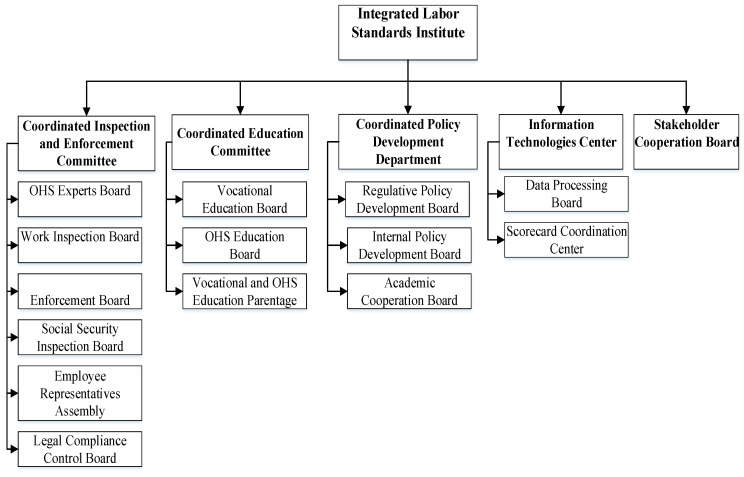
Organizational Chart of the Proposed Integrated Labor Standards Institute.

Institutional system improvement requirements call for an institution to coordinate and control the labor standard practices in the Turkish construction industry. This institution should be a national state-governed public institution with regulation, inspection, and enforcement tasks, or a private sector company, providing insurance and consulting. Since the stakeholder analysis concluded the strong influence of the state, and the literature review and expert interviews revealed the immaturity of the construction industry in Turkey, we omitted the private sector-dominated option in the proposed institutional system design. Figure 7 shows the organizational chart of the designed “Integrated Labor Standards Institute”.

Figure 7 and Table 5 list this institutional system’s units by the related labor standards, principal roles, and the associated innovations introduced in the system design.

The proposed organizational structure for an Integrated Labor Standards Institute is structured based on the occurring themes and needs from the AS-IS analysis (Section 3.1), regarding the problems related to OHS expertise, work inspections, enforcement mechanisms, and education (the lack of qualified workforce). Such an institution is supposed to have committees, a department, an information technology (IT) center, and a Stakeholder Cooperation Board. The Coordinated Inspection and Enforcement Committee should have boards of OHS Experts, Work Inspection, Enforcement, Social Security Inspection, and Legal Compliance Control, and also an assembly formed by employee representatives offering an inclusive perspective to the committee. The Coordinated Education Committee should deal with vocational and OHS education through its boards. Additionally, the formal collaboration of the Turkish construction industry with universities, governmental policy institutions, and labor unions is low [12], causing their low influence on the institutional system of labor standards, as revealed by the stakeholder analysis. As a response to this need, a Coordinated Policy Development Department is recommended to have regulative policy development and university collaboration boards. For meeting the IT infrastructure, services, and operations needs of the proposed scorecard application, the institution should also have an advanced IT function, working on data processing of the collected information from the companies and institutions. The stakeholder analysis showed that the cooperation between stakeholders and the management of stakeholders in the institutional system has significant deficiencies. Hence, the institution should also host a Stakeholder Cooperation Board.

As shown in Table 5, the OHS Experts Board has the main role of auditing and guidance while the Work Inspection Board aims to prevent political interventions and pressures by providing the autonomy of the proposed institution. More importantly, the Work Inspection Board should aim to increase information channels between OHS experts and employee representatives for disseminating knowledge and information as valuable feedback from experts to the operational context. The Social Security Inspection Board also serves the same need. The Enforcement Board should be authorized for enforcement implementation. In addition, it should determine the level of fines based on the profitability of operations with non-conforming labor standards. The Employee Representative Assembly is the reflection of the inclusiveness of the institution providing feedback and consultation to all units. Education Boards should act on educational policies, and the planning and implementation of educational services in accordance. Vocational and OHS Education Parentage’s function is critical for providing information and knowledge transfer between companies in the industry, and a mechanism for collaborative learning and improvement. The Regulative Policy Board is also another needed function for providing advocacy of the system’s needs in regulative changes and also for disseminating knowledge about regulations to all stakeholders.

#### 3.2.5. Scorecard Application Design as a Performance Assessment Tool

**Figure 8:**
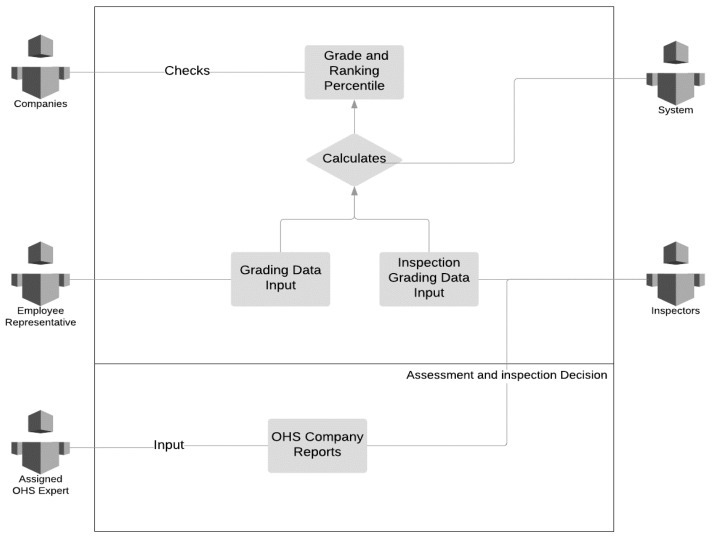
Use Case Diagram.

A scorecard application is designed as an enforcement tool to motivate profit-oriented construction companies towards elevating their labor standards to be utilized in bidding evaluations and incentive mechanisms. The expert from the Health and Safety Labor Watch of Turkey claimed the need for such a scorecard. Each company’s OHS performance and violation history can be monitored and evaluated by the inspectors and project owners. The scorecard application is built to collect, process, and redeliver the data supplied by the Inspection, Enforcement, and Education Boards to all units and stakeholders. In the public and private sector partnerships and contracting, this score should be referred to and play a crucial enforcement role in contractor selection and approval in public bidding. A use case diagram (Figure 8) was compiled to illustrate the scorecard application’s running mechanism. Use case diagrams are useful for the representation of the actors and use cases of a system, in which the actors are the environment of the modeled system and use cases form the system in operation [98]. As Figure 8 presents, the scorecard application has companies, employee representatives, OHS experts, inspectors, and the software system for grade calculation as “actors”. It should enable the grading of the companies’ labor standards performance on selected criteria of institutional system analysis, namely working hours, employee services, job security, OHS, and vocational education.

Employee representatives, inspectors, OHS experts, and education boards provide data within periods set by the Employee Representatives’ Assembly. The OHS Education Board and Vocational Education Board are responsible for periodically conducting tests to measure its vocational and OHS training levels. The Data Processing Board gives construction companies’ final rates, average grades, and assigned percentiles. In Turkey, large-scale construction projects operated by public–private partnerships (PPPs) amounted to USD 140 billion in 2018 [99]. Being subcontractors in such projects, construction companies can be motivated to obtain high scores from the scorecard. In this context, the scorecard application enables the integration of labor standards performance of companies into their business cases.

## 4. Discussion

Previous research mainly focused on particular employment regimes and OHS problems in Turkish construction, identifying some insufficiencies in the regulation, inspection, and enforcement systems, as discussed in the literature review section. We selected the criteria used in this study in accordance with the topics that were addressed in previous literature: “Working Time and Rest” [1,48]; “Job Security” [13,48,49,52,53]; “OHS” [14,47,56,59,64]; “Education” [48,49]; and “Employee Services” [48,50,56]. Based on the previous theoretical background, our study methodologically analyzed the current system and proposed an institutional system design that could holistically address the multi-layered and interlinked labor standard and OHS problems in the sector.

The analysis revealed various problems in the inspection and monitoring systems of OHS regulations in the Turkish construction sector. The current system does not have a sufficient number of inspectors for adequate inspection. In 2017, only 8% of the employees were inspected and 30% of the investigated occupational accidents were in the construction sector [89]. Insufficient penalties and weak inspection and enforcement mechanisms contribute to the high accident and fatality rates. The high probability of accidents in construction activities is related to the high rate of micro and small-scale enterprises (<49 employees) [100] which account for 97% of employment [89]. Employing seasonal, migrant, and self-employed workers under temporary conditions, these companies practically do not attach sufficient importance to OHS. Although it is obligatory to employ part-time OHS specialists and physicians in small workplaces, the duration and quality of these services are not sufficient [89].

Currently, there is no restriction to becoming a construction contractor in Turkey. Moreover, despite the certification requirement for seeking employment as a construction worker, around 30% of employees work without any certification [101]. The OHS training and use of individual protective equipment remain insufficient [12,102] because of both employees’ ignorance and time pressure as well as employers’ cost concerns [103]. Weaknesses in OHS management [104] are especially relevant in small businesses [105]; however, considering that large construction firms outsource work to many subcontractors, risks are huge even in large companies [106]. According to the legislation, the principal employer could outsource a job to a subcontractor either if it is an auxiliary job rather than a part of the main job or the job requires technological expertise. As Yılmaz [107] emphasizes, in practice, in large-scale building projects, almost every part of the construction product is divided into pieces and then outsourced to various subcontractors. The OHS legislation grants subcontractor companies the freedom to receive OHS services independently. Assigning OHS professionals and applying OHS processes separately for each subcontractor causes almost uncontrollable chaos on construction sites. Considering the widespread principal–subcontractor relations, there is a need for specific OHS legislation for the building sector [107].

Akgül and Doğan [12] emphasize the need for collaboration among the industry, government, and universities to increase the level of readiness of civil engineering and construction technician students, who are expected to have significant responsibility, to reduce occupational accidents. The stakeholder analysis showed that the ILO, universities, labor unions, NGOs, and municipalities have a “low” influence on the institutional system of labor standards and OHS in the Turkish construction sector. The low influence of the unions can be explained by the significantly low unionization rate. In addition, the prevalent micro and small-size firms are not able to use scientific and systematic methods. They do not, therefore, attempt to contact universities (as stated in the expert interview with the union representative), causing the low influence of universities in the system. Additionally, there exist limited studies about the labor standards in the construction industry from a holistic perspective, and most of the publications (that were not cited in this paper) are about technical solutions to work safety. From the limited theoretical background, it can be understood that universities are not involved in the processes and interactions of the institutional system of labor standards in the Turkish construction sector. Moreover, OHS specialists’ influence was also found to be unexpectedly low. Despite the laws precipitating compulsory OHS training and precautions, occupational safety has not yet been established as a culture in Turkey. The inadequacy of the training received by OHS specialists and the problems they encounter on construction sites are addressed in the literature [88,108]. Under such conditions, OHS specialists experience difficulties while providing training and producing solutions for failures, including regulation and inspection system deficiencies. Aligned with the literature findings [59,61,88,89], given the limited power of OHS specialists and inspectors due to their minimal authority and entitlement challenges, the institutional system remains inefficient regarding the close monitoring and enforcement of OHS incidents and non-conformances. This gap causes governance and impact/enforcement weaknesses in the institutional system. However, the state has a strong influence on Turkey’s institutional labor standards system, supporting the remarks of the ILO [2] and Usmen and Baradan [62]. Hence, the proposed system model attempted to utilize this powerful role of the state in establishing an institution and forcing/directing construction companies and value chain players to use the proposed institutional, informative systems actively.

The regulative analysis showed a significant gap in the working time and employment security of Turkish construction workers compared to the standards of the ILO and EU. Social security problems in the sector were commonly addressed by previous research [48,49,89]. The literature referred to subcontracting regimes as a root cause of OHS, job security, wage, working hour, and education problems [52]. Falling behind with 14 vacation days/year and an OECD EPL score of 2.33 [84], Turkey’s legislation is against individual and collective dismissals, providing evidence of the sector’s social security problems and underlining the urgent need for job security and working time regulations. Though the official weekly working hours are balanced with international standards, this limit is often exceeded due to the lack of inspection and enforcement, as the interviews revealed.

The analysis of workflows in the institutional system showed that the violations and errors in practice quickly go unnoticed or go without enforcement. The current system fails in the lack of inspection, review of complaints, and lack of governors’ action or enforcement. Authorized institutions and their regulation and coordination are essential components of industrial development [109,110], which is an issue also covered by the United Nations’ SDG No. 16 [20]. By the literature findings [48,56,59], execution of an adequate inspection system and enforcement mechanisms stands as one of the root causes of non-compliant labor management and OHS practices. Under this topic, the cause-and-effect analysis revealed a lack of initial control mechanisms, bureaucratic and political barriers, a lack of inspection system continuity, and low fines and penalties. The study shows that the high turnover rate in the sector is another root cause. Guthrie [93] concludes that high turnover rates result in low productivity, forcing employers to take strict, sometimes extreme, labor control regimes and causing violations of labor laws (such as precarious work or unpaid extended work hours). Requirements analysis also pointed to the need for pre-operational control, fair fines, and governmental grants, which previous studies rarely mentioned, contributing to the theoretical knowledge.

The main component of the proposed labor standards and OHS institutional system design for the Turkish construction industry is an institution that integrates and links the stakeholders. This institutional design is expected to meet the coordination, communication, involvement, and awareness raising needs among stakeholders for responding to the labor and OHS problems and possible solutions, as offered as an improvement area by the ILO [2]. This structure functions as a “Standards Institute” governed by the state that coordinates inspection and enforcement, education, policy development, and information management activities through a Stakeholder Cooperation Board. Such structures can fill the “inclusive institutions” gap [109,110] and meet the systematic institutional system improvement needs. This study is also an example of practices reflecting the ILO instruments relevant to UN SDG No. 16 and 17 [20].

Another design component is a scorecard application tracking the labor standards practices and OHS performances of construction companies on several criteria such as work hours, employee services, job security, OHS, and vocational education. This design component shares a similar perspective with the work of Kang et al. [111] which offers a plan to motivate construction companies in South Korea for accident prevention activities. The scorecard application corresponds to the need for a better system to keep track of incidents and accidents in the OHS domain, as recommended by Human Resources Development Convention no. 142 of the ILO (1975) [73] at a global level and by Duman and Etiler [56] in the Turkish construction industry context. In the competition for PPP projects, labor standards and OHS scores from this application can accompany price competitiveness in bidding.

**Figure 9:**
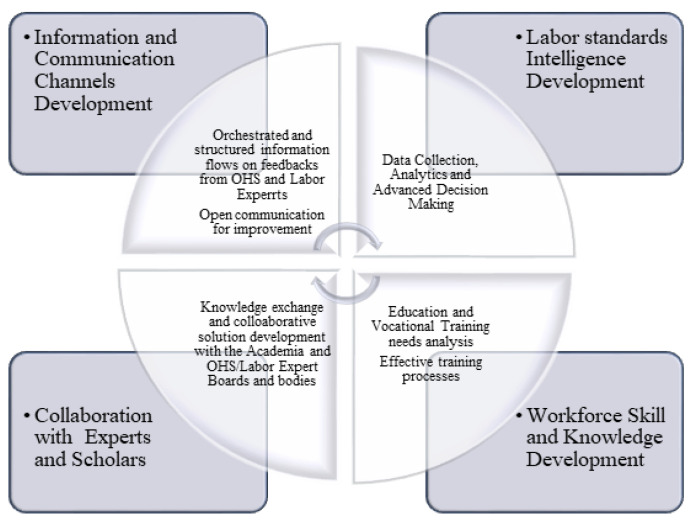
Recommended Strategic Agenda for the Policy Makers and Employers in the Construction Industry.

The findings also provided some strategic highlights and improvement points for the industry. The industry leaders, OHS managers or practitioners, and labor standards experts can utilize the findings from the study in their OHS and labor standards-related roadmapping and strategy-making processes. In addition, the findings can be helpful in the advocacy processes of industry representatives toward regulative improvements. Based on the analysis and proposed solutions from the study, a strategic agenda is recommended to industry decision-makers in Figure 9. The roadmap consists of proposed steps toward improved information and communication channels, collaboration, data analytics, and education/training processes in the construction sector.

## 5. Conclusions

In the construction industry, the employees’ conditions have worsened globally by the cost-effectiveness strategies causing unbalanced employment regimes. The sector became a leader in occupational accidents accompanied by the pervasive problems of low wages, long working hours, and limited social security inclusion. There is a need for holistic, multi-dimensional, and systematic institutional system change to improve efficiency, coordination, and inspection mechanisms in the Turkish construction industry which has structural labor standard problems and high fatality rates due to the subcontracting regime and regulation implementation failures.

To contribute to the literature on the institutional systems in enhancing labor standards, this article analyzed the current situation and presented a system proposal for improving the Turkish construction industry’s work conditions as a case study on highly hazardous industries. We combined system analysis and design methods with labor studies by a multi-disciplinary approach. The proposed system is expected to provide a roadmap for policymakers and regulative bodies who work on systemic changes at the institutional and operational levels to improve labor conditions in the construction sector. As the components of the institutional system, we holistically considered and analyzed all stakeholders, information flows, causes, and effects of problems and then offered an update to labor legislation by proposing an institution and scorecard application design in the Turkish construction industry. The application of the system design methods such as root cause analysis, QFD, and use case development is proved to be valuable and practical in political–institutional design. The system proposal could be applied to other countries that follow the low-road strategy such as South Korea, the UK, the USA, Australia, and Spain [4] where achieving construction safety and reducing worker fatal injury rates are still challenging due to systemic failures [111,112,113,114,115,116]. The proposed system for Turkish construction provides an avenue for future research for the examination of the degree of similarity with the construction sectors of other low-road-taking countries and an assessment of the adaptability of the proposed system to other country contexts, which is beyond the scope of this study.

The findings from the literature review, content analysis, and in-depth interviews with the experts revealed a need for additional institutional arrangements to establish a collaborative and sustainable environment in the Turkish construction sector. For this purpose, we applied system analysis and design methods for exploring the problems and proposing solutions for improving the current institutional system of labor standards. The current system is analyzed regarding regulations, inspection and enforcement, working hours, employee services, job security, OHS, and education. The regulatory analysis showed that Turkey’s existing labor regulations are compatible with the EU and ILO; however, they have been hardly put into practice in the Turkish construction industry. In terms of all the chosen labor standard criteria, sector realities have been illegitimate. For example, the industry’s average working hours have been above the legal minimum limit; the required employee services have been non-existent or inadequate. Inspected site rate is low, primarily due to the limited number of inspectors employed in the inspection and enforcement system. In addition, in most cases, the fines are less costly than the investments and the operations required for establishing higher labor standards in work sites.

By a cause-and-effect analysis, the study identified the root causes of the chronic problem of low labor standards in the sector: Deficiencies in the institutional system such as a lack of initial control and continuous inspection mechanisms, bureaucratic and political barriers, low fines, and lack of government grants. In addition, excessive work hours and insufficient employee services, OHS, job security, and vocational education caused labor standard problems in workplaces.

Limitations and further research: Although the validation of the proposed model was fulfilled in the study by a QFD intervention matrix, verification of such a system is only possible after the actual implementation of this system in real life, which is beyond the scope and capabilities of a logical system design study. In further phases of the research and practicing the proposed system, an evident feasibility analysis based on actual application results can robustly verify the designed system. Furthermore, as a revealed root cause of labor standard problems, the “immaturity of the industry” was excluded from the design proposal due to the immense scope of this problem. Some further exploration can elaborate on the impact of industrial immaturity on labor standards. Additionally, construction sector employers could not be interviewed since they rejected our requests. Some collaborative research settings with employers’ unions can help to complement the employers’ views in determining the root causes of the problem. Delphi techniques aiming at consensus building among experts could be utilized for methodological improvements, and multi-criteria decision-making models such as AHP or TOPSIS could provide more consistent results from expert interviews and ratings.

## Figures and Tables

**Table 1:** Interviewee Profiles.

Interviewees	Years of Experience	Scope
Labor law expert	15	Labor standards and Labor Act of Turkey
OHS expert	17	Certified by the Ministry of Labor and Social Security; experience in the field and OHS consultancy
OHS expert	12	Certified by the Ministry of Labor and Social Security; experience in the field
Worker representative from the labor union	14	Construction worker-contracted
Health and Safety Labor Watch representative	21	OHS expertise; Joint Health and Safety Unit (Organized Industry Region) labor watch manager
Inspection and enforcement expert	15	OHS Institution—Republic of Turkey Ministry of Labor and Social Security
Inspection and enforcement expert	14	OHS Institution—Republic of Turkey Ministry of Labor and Social Security

**Table 2:** Roles and Responsibilities of Sector Stakeholders.

Stakeholder	Affected Criteria	Main Roles/Responsibilities	Influence
Construction Firms	All Criteria	♦ Provide the required labor standards	Medium
		♦ Increase workers’ competency through training	
Employees	All Criteria	♦ Have knowledge about labor rights	Medium
		♦ Inform state departments when illegal practices occur	
		♦ Refuse to implement any illegal practices	
Governors	OHS, Protection	♦ Carry out instructions of work inspectors	
Grand National Assembly of Turkey	All Criteria	♦ Makes laws about labor standards	High
		♦ Establishes commissions that investigate and provide ♦ solutions to deficiencies in labor standards	
Institute of Occupational Health and Safety	OHS	♦ Examines OHS practices in workplaces	Medium
		♦ Prepares projects that help to ensure OHS	
International Labor Organization	All Criteria	♦ Promotes internationally recognized labor rights	Low
		♦ Conducts research related to labor standards	
		♦ Designs projects and arranges conventions to improve coordination for advanced labor standards	
Labor Unions	All Criteria	♦ Assist in raising labor standards via collective bargaining	Low
		♦ Conduct researches and inform the public about current issues	
		♦ Increase awareness among workers	
Media	All Criteria	♦ Informs the public and raises awareness of labor standards issues	Low
		♦ Provides communication channels	
Ministry of Family, Labor and Social Services	All Criteria	♦ Regulates and control work conditions	High
		♦ Maintain the effectiveness of the social security system	
Municipalities	All Criteria	♦ Give permission to construction sites	Low
Non-governmental Organizations	All Criteria	♦ Raise awareness of labor standards	Low
		♦ Provide communication channels and increase coordination ♦ to improve labor standards	
Occupational Health and Safety Specialists	OHS	♦ Oversee workplace health and safety	Low
Occupational Qualification Institution	Education	♦ Designs the certification processes	Medium
		♦ Ensures the sufficiency of workers’ vocational education	
		♦ Provides courses, seminars and materials for vocational education	
Presidency of the Republic of Turkey	All Criteria	♦ Establishes relevant ministries to ensure necessary labor standards	High
Social Policies Board	Job Security	♦ Develops policy recommendations for low-income population	Medium
Social Security Institute	Job Security	♦ Conducts social security policies	Medium
		♦ Carry out coordination and cooperation between public administrations in the field of social security	
		♦ Increases awareness of social security rights	
Universities	All Criteria	♦ Conduct research on labor standards problems	Low
		♦ Develop solution-oriented projects	
Work and Social Security Training&Research Center	Education	♦ Provides training, research, publication, documentation and consultancy on working life and social security issues	Medium
		♦ Collects and publishes statistics on labor standards	
Work Inspection Institute	Work hour, Job Protection, OHS	♦ Examines construction sites in terms of relevant labor standards and initiates enforcement procedures in case of violations	Medium

**Table 3:** Comparison of Findings from Literature, Regulative Analysis, and Interviews for Triangulation.

Criteria	Themes from Literature Content Analysis	Themes from Regulative Analysis	Themes Coded from Interviews	Themes Occurred from the Coded Interviews	Interviewee’s Concordance	Rater’s Concordance
(1)(2)(3)(5)		-		Lack of initial control mechanisms	3/7	3/4 = 75%
(1)(2)(3)(5)		-		Bureaucratic/political pressures	4/7	2/4 = 50%
(1)(2)(3)(5)	Insufficient/low fines and penalties enforcing execution	-	Insufficient low fines and penalties enforcing execution		7/7	4/4 = 100%
(1)(2)(3)(5)	Lack of continuous inspection mechanisms	-	Lack of continuous inspection mechanisms		4/7	4/4 = 100%
(1)(2)(3)(4)(5)	Immaturity of the sector	-	Immaturity of the sector		5/7	4/4 = 100%
(1)(2)(3)(4)(5)		-		Lack of governmental grants and incentives for labor standards	4/7	3/4 = 75%
New		-		Low prestige of construction blue-collar jobs	3/7	3/4 = 75%
(5) Employee Services	Insufficient employee services	Sufficient in Turkey’s regulations	Insufficient employee services		7/7	4/4 = 100%
(4) Education and Vocational Training	Insufficient vocational education	Sufficient in Turkey’s regulations	Insufficient vocational education		6/7	4/4 = 100%
(2) Job Security & Registered working	Job insecurity	Insufficient in Turkey’s regulations	Job insecurity		6/7	4/4 = 100%
(1) Working and Rest Time	Excessive working hours	Insufficient in Turkey’s regulations	Excessive working hours		4/7	3/4 = 75%
(3) OHS	Insufficient OHS practices	Sufficient in Turkey’s regulations	Insufficient OHS practices		7/7	4/4 = 100%

**Table 4:** Root Causes and Design Requirements.

Main Causes	Root Causes	Design Specifications
Ineffective Inspection and Enforcement System	Lack of Initial Control Mechanisms	Effective Pre-occupational Control Mechanism
	Bureaucratic and Political Barriers	The Objectivity of the Inspection and Enforcement System
	Low Fines	Fair Fines
	Lack of Continuous Inspection Mechanisms	Effective Continuous Inspection System
High Turnover Rates	Excessive Working Hours	Adequate Working Hours
	Insufficient Employee Services	Adequate Employee Services
	Insufficient OHS	Adequate OHS
	Insufficient Vocational Education	Adequate Vocational Education
	Insufficient Job Security	Adequate Job Security
Low Investment in Labor Standards	Lack of Governmental Grants	Proper and Fair Governmental Grants
	Immature Sector	

**Table 5:** The Proposed Organization’s Main Roles and Innovative Actions.

Unit	Related Labor Standard	Main Role	Improvements
OHS Experts Board	OHS	Audit and Guidance	♦ OHS specialists are assigned to construction firms as observers/guiders/auditors of the institute. They are no longer employed by the employer.
Work Inspection Board	Working Hours, Employee Services, OHS	Audit	♦ Political pressures reduced by the autonomous institution ♦ Increased information channels by OHS experts and employee representatives
Enforcement Board	Working Hours, Employee Services, OHS, Job Security	Determination and Implementation of Enforcement	♦ Enforcement authority is given. ♦ It determines fines by calculating the profit from improper practices
Social Security Inspection Board	Job Security	Audit	♦ Increased information channels by OHS experts and employee representatives
Employee Representatives’ Assembly	Working Hours, Employee Services, OHS, Job Security, Education	Audit, Feedback, Consultation	♦ Provides information to audit, planning, cooperation and consulting units
Legal Compliance Control Board	Working Hours, Employee Services, OHS, Job Security	Audit	♦ It implements pre-operational legal compliance control in terms of OHS. ♦ It is necessary to get approvals for construction projects.
Vocational Education Board	Vocational Education	Education Policy Planning and Implementing	♦ Increased information channels by Data Processing Board ♦ Increased effectiveness by Scorecard
OHS Education Board	OHS	Education Policy Planning and Implementing	♦ Increased information channels by Data Processing Board. ♦ Increased effectiveness by Scorecard
Vocational and OHS Education Parentage	Vocational Education, OHS	Education	♦ According to the results obtained from the scorecard, the firms with a high number of relevant training points provide training to the lower ones. If the company with a low score develops itself and does not fall below a certain point for a specific period of time, the training provider earns points and grants.
Regulative Policy Development Board	Working Hours, Employee Services, OHS, Job Security, Vocational Education	Consultation	♦ Offers legal updates to the legislators about problems related to the construction sector regulations.
Internal Policy Development Board	Working Hours, Employee Services, OHS, Job Security, Vocational Education	Planning	♦ It performs internal planning for the Institute according to the data and recommendations from other units.
Collaboration with Scholars Board	Working Hours, Employee Services, OHS, Job Security, Vocational Education	Consultation	♦ Provides information and advice sharing with academicians
Data Processing Board	Working Hours, Employee Services, OHS, Job Security, Vocational Education	Data Supply	♦ Stores data from all units ♦ Processes data ♦ Supplies processed data to related units
Scorecard Coordination Center	Working Hours, Employee Services, OHS, Job Security, Vocational Education	Data Supply	♦ Implementing and maintaining Scorecard Application ♦ It provides related data to related units
Stakeholder Cooperation Board	Working Hours, Employee Services, OHS, Job Security, Vocational Education	Cooperation and Consultation	♦ Improves coordination among stakeholders

## Data Availability

Not applicable.

## References

[1] International Labour Organization (2001). The Construction Industry in the Twenty-First Century: Its Image, Employment Prospects and Skill Requirements.

[2] International Labour Organization (2015). Good Practices and Challenges in Promoting Decent Work in Construction and Infrastructure Projects. Proceedings of the Paper for Discussion at the Global Dialogue Forum, Sectoral Policies Department.

[3] Lew Y.L., Lai S., Toh T., Tan O., Felicia Y., Yow L. (2020). Quality performance of multi-layered subcontracting practices in Malaysian construction industry. IOP Conf. Ser. Earth Environ. Sci..

[4] Bosch G., Philips P. (2003). Building Chaos: An International Comparison of Deregulation in the Construction Industry.

[5] KPMG Construction—Sectoral Overview 2018. https://assets.kpmg.com/content/dam/kpmg/tr/pdf/2018/01/sektorel-bakis-2018-insaat.pdf.

[6] International Labour Organization (2005). Facts on Safety at Work.

[7] Hino Y., Ohdo K., Takanashi S., Takahashi H. (2011). International survey on prevention system of labor accidents at the construction site. Procedia Eng..

[8] Turkish Statistical Institute (2017). Labour Force Status of Population. http://www.turkstat.gov.tr/PreIstatistikTablo.do?istab_id=2262.

[9] Republic of Turkey Ministry of Labor and Social Security Communique on Numbers of Workers and Union Members. Official Gazette No.: 31900 and dated 22 January 2022. https://www.csgb.gov.tr/istatistikler/calisma-hayati-istatistikleri/sendikal-istatistikler/isci-sayilari-ve-sendikalarin-uye-sayilari-hakkinda-tebligler/.

[10] Karaman A.E., Çivici T., Kale S. İşçi Sağlığı ve İş Güvenliğinin İnşaat Sektöründeki Yeri ve Önemi. Proceedings of the 3rd Occupational Health and Safety Symposium.

[11] Öçal M., Çiçek Ö. (2017). Comparative analysis of occupational accident data in Turkey & EU. HAK-IS Int. J. Labour Soc..

[12] Akgül M., Doğan Y. (2020). Awareness analysis on occupational health and safety in the construction sector: Sample of Marmara and Central Anatolian regions. Eng. Sci..

[13] Duman E., Hamzaoğlu O. (2011). Impression of work accidents of employees at a construction site in Istanbul. Turk. Med. Assoc. J. Occup. Health Saf..

[14] Gürcanlı G.E. (2013). Focusing on deregulated and despotic labor regime: Health and safety in the construction industry. Eğitim Bilim Toplum.

[15] Bilir E. Sosyal Güvenlik Kurumu Dalga mı Geçiyor? 24 February 2013. http://www.sendika.org/2013/02/sosyal-guvenlik-kurumu-dalga-mi-geciyor-ertugrul-bilir/.

[16] Gültekin-Karakaş D., Yusufi F., Hisarcıklılar M. (2021). An evaluation of labor standards in the Turkish construction industry from the perspective of sectoral development. Mülkiye J..

[17] Mızrak K.C., Tolon M. (2017). Occupational Health and Safety and Sustainable Development in Turkey in the construction industry. Nişantaşi Univ. J. Soc. Sci..

[18] International Labour Organization (2013). Decent Work Indicators.

[19] International Labour Organization (2019). Rules of the Game: An Introduction to the Standards-related Work of the International Labour Organization.

[20] United Nations Do You Know All 17 SDGs?. https://sdgs.un.org/goals.

[21] International Labour Organization (2020). 2030 Agenda for Sustainable Development. https://libguides.ilo.org/2030-agenda-en/standards.

[22] Debrah Y.A., Ofori G. (2001). Subcontracting, foreign workers and job safety in the Singapore construction industry. Asia Pac. Bus. Rev..

[23] Mayhew C., Quinlan M., Ferris R. (1997). The Effects of subcontracting/outsourcing on occupational health and safety: Survey Evidence from four Australian industries. Saf. Sci..

[24] Perrin B. (2013). Evaluation of Payment by Results (PBR): Current Approaches, Future Needs.

[25] Valluru C.T., Dekker S., Rae A. (2017). How and why do subcontractors experience different safety on high-risk work sites?. Cogn. Technol. Work.

[26] Lingard H., Rowlinson S. (2005). Occupational Health and Safety in Construction Project Management.

[27] Lingard H. (2013). Occupational health and safety in the construction industry. Constr. Manag. Econ..

[28] Mustchin S. (2014). Union modernisation, coalitions and vulnerable work in the construction sector in Britain. Ind. Relat. J..

[29] Mayhew C., Quinlan M. (1997). Subcontracting and occupational health and safety in residential building industry. Ind. Relat. J..

[30] Byrne J., van der Meer M. (2000). The Construction Industry in Spain: Flexibilisation and Other Corporatist Illusions. Proceedings of the International Conference on Structural Change in the Building Industry’s Labour Market, Working Relations and Challenges in the Coming Years, Institut Arbeit und Technik.

[31] Wong F., So L. Restriction of the Multi-Layers Subcontracting Practice in Hong Kong—Is it an Effective Tool to Improve Safety Performance of the Construction İndustry?. Proceedings of the CIB Conference 2002.

[32] Azari-Rad H., Philips P., Thompson-Dawson W. Subcontracting and injury rates in construction. Proceedings of the Industrial Relations Research Association, Proceedings 2003 (55th Annual Meeting).

[33] Loosemore M., Andonakis N. (2007). Barriers to implementing OHS reforms—The 4 experiences of small subcontractors in the Australian construction industry. Int. J. Proj. Manag..

[34] Chiang Y.H. (2009). Subcontracting and its ramifications: A survey of the building industry in Hong Kong. Int. J. Proj. Manag..

[35] Yung P. (2009). Institutional arrangements and construction safety in China: An empirical examination. Constr. Manag. Econ..

[36] Wells J. (1996). Labour migration and international construction. Habitat Int..

[37] Pattanaik B. (2009). Young migrant construction workers in the unorganised urban sector. South Asia Res..

[38] Underhill E., Quinlan M. (2011). How precarious employment affects health and safety at work: The case of temporary agency workers. Ind. Relat..

[39] Yea S. (2015). Trafficked enough?. Missing bodies, migrant labour exploitation, and the classification of trafficking victims in Singapore. Antipode.

[40] Tutt D., Pink S., Dainty A.R.J., Gibb A. (2013). In the air and below the horizon: Migrant workers in UK construction and the practice-based nature of learning and communicating OHS. Constr. Manag. Econ..

[41] Buckley M., Zendel A., Biggar J., Frederiksen L., Wells J. (2016). Migrant Work and Employment in the Construction Sector.

[42] Wells J. (2007). Informality in the construction sector in developing countries. Constr. Manag. Econ..

[43] Wickramasekara P. (2015). Bilateral Agreements and Memoranda of Understanding on Migration of Low Skilled Workers: A Review.

[44] International Labour Organization Global Trends on Occupational Accidents and Diseases. World Day for Safety and Health at Work 28 April 2015..

[45] Eurostat Accidents at Work Statistics 2020. https://ec.europa.eu/eurostat/statistics-explained/index.php/Accidents_at_work_statistics.

[46] Turkish Statistical Institute (2018). Turkish Social Security Institution Statistical Almanac. http://www.sgk.gov.tr/wps/portal/sgk/tr/kurumsal/istatistik/sgk_istatistik_yilliklari.

[47] Gürcanlı G.E. (2013). Analysis of deaths and injuries in the construction industry. Turk. Med. Assoc. J. Occup. Health Saf..

[48] Koçak H. (2013). Structure of employment in construction and characteristics of the labor regime. Turk. Med. Assoc. J. Occup. Health Saf..

[49] INTES (The Turkish Employers’ Association of Construction Industries) Construction Sector Report, September 2018. https://enerji.mmo.org.tr/wp-content/uploads/2019/05/%C4%B0N%C5%9EAAT-SEKT%C3%96R-RAPORU.pdf.

[50] Çınar S. (2018). New actors and new conflicts in the construction sector: Syrian workers from the perspective of local workers. Labour Soc..

[51] Harroff-Tavel H., Alix N. (2013). Tricked and Trapped Human Trafficking in The Middle East.

[52] Kara M.A., Kurtulmuş M.M. (2015). Field study on the employment of migrant workers in the construction industry. DISKAR.

[53] Şahin Ç. (2014). Türkiye’de Göçmen İşçiler Sorunu. Toprak Employer’s Union of Turkey. Toprak İşveren E-J..

[54] Uzunkaya M. (2013). Uluslararası Rekabet Edebilirlik Çerçevesinde Türk İnşaat Sektörünün Yapısal Analizi.

[55] Gürbüz H., İbrakovic H. (2017). Work safety, safety performance and safety culture in businesses. J. Soc. Sci..

[56] Duman E., Etiler N. (2013). Construction sector and worker health. Turk. Med. Assoc. J. Occup. Health Saf..

[57] Baradan S., Dikmen S.Ü., Müngen U., Aytekin O., Sönmez G. (2011). Türkiye’deki iş sağlığı ve güvenliği hizmetleri mevzuatının inşaat sektörü açısından incelenmesi. Türkiye Mühendislik Haberleri.

[58] Akbıyıklı R., Dikmen Ü.S. (2018). Main indicators of OHS management in construction sites. Düzce Univ. J. Sci. Technol..

[59] Yılmaz F. (2015). Statistical evaluation of occupational health and safety inspections in Turkey. ISGUC J. Ind. Relat. Hum. Resour..

[60] Labor Inspection Board of Republic of Turkey Ministry of Labor and Social Security (2017). 2016 Yılında İş Sağlığı ve Güvenliği Yönünden Gerçekleştirilen Programlı Teftişlerin Sonuç Raporu Özetleri.

[61] Akboğa K.Ö., Baradan S., Gurcanli G.E., Dikmen Ü., Bayram İ. İş güvenliği uzmanlığı: Sistemin işleyişinin değerlendirilmesi üzerine bir araştırma çalışması. Proceedings of the 5th Workers’ Health and Occupational Safety Symposium.

[62] Usmen M., Baradan S. (2011). Factors affecting improvements in occupational health and safety in the construction industry: The case of the USA. Türkiye Mühendislik Haberleri Derg..

[63] Baradan S. (2006). An overview of occupational safety and health culture in the construction industry in Turkey and comparisons with developed countries. Dokuz Eylül Univ. Eng. Fac. J. Sci. Eng..

[64] Gürcanlı G.E., Müngen U. (2013). Analysis of construction accidents in Turkey and responsible parties. Ind. Health.

[65] Kvale S. (1983). The qualitative research interview: A phenomenological and a hermeneutical mode of understanding. J. Phenomenol. Psychol..

[66] Systems Engineering Body of Knowledge (SEBoK) (2021). Guide to the Systems Engineering Body of Knowledge. SEBoK Version 2.5..

[67] Schmeer K. (2001). Stakeholder Analysis Guidelines. https://watsanmissionassistant.org/wp-content/uploads/2018/08/stakeholder-analysis-guidelines-unknown.pdf.

[68] Varvasovszky Z., Brugha R. (2000). How to do (or not to do) a stakeholder analysis. Health Policy Plan..

[69] Šaloun P., Malčik M., Andrešič D., Nespěšný D. (2018). Using Eyetracking to Analyse How Flowcharts are Understood. Proceedings of the 2017 IEEE 14th International Scientific Conference on Informatics.

[70] Ishikawa K., Loftus J.H. (1991). Introduction to Quality Control.

[71] Desai M.S., Johnson R.A. (2013). Using a fishbone diagram to develop change management strategies to achieve first-year student persistence. SAM Adv. Manag. J..

[72] Hauser J.R., Clausing D. The House of Quality. Harward Business Review 1988. https://hbr.org/1988/05/the-house-of-quality.

[73] International Labour Organization (2017). NORMLEX Information System on International Labour Standards. https://www.ilo.org/dyn/normlex/en/f?p=NORMLEXPUB:12030:0::NO.

[74] Organization for Economic Co-operation and Development (2005). Compare Your Country Employment Protection Legislation. https://www1.compareyourcountry.org/employment-protection-legislation?cr=oecd&lg=en&page=0.

[75] Allard G.J. (2005). Measuring job security over time: In search of a historical indicator for EPL (Employment Protection Legislation). Instituto de Empresa Business School Working Paper No. WP05-17.

[76] United Nations Educational, Scientific and Cultural Organization (UNESCO) (2003). Technical and Vocational Education and Training for The Twenty-First Century: UNESCO and ILO Recommendations.

[77] International Labour Organization (2009). Worker’s Housing. https://www.ilo.org/wcmsp5/groups/public/---ed_emp/---emp_ent/---multi/documents/publication/wcms_116344.pdf.

[78] Herzberg F. (1966). Work and the Nature of Man.

[79] International Labour Organization (2006). Strategies and Practice for Labour Inspection.

[80] Labor Act of Turkey Law No. 4857, Date of Enactment 22 May 2003. Official Gazette Dated 10 June 2003 and No. 25134. https://www.mevzuat.gov.tr/MevzuatMetin/1.5.4857-20140910.pdf.

[81] International Labour Organization C047-Forty-Hour Week Convention, 4 June 1935, Information System on International Labour Standards. https://www.ilo.org/dyn/normlex/en/f?p=NORMLEXPUB:12100:0::NO::P12100_ILO_CODE:C047.

[82] European Union Council Directive 2003/88/EC of the European Parliament and of the Council of 4 November 2003 Concerning Certain Aspects of the Organisation of Working Time. Access to European Union Law. http://data.europa.eu/eli/dir/2003/88/oj.

[83] International Labour Organization C132-Holidays with Pay Convention, 24 June 1970, Information System on International Labour Standards. https://www.ilo.org/dyn/normlex/en/f?p=NORMLEXPUB:12100:0::NO::P12100_ILO_CODE:C132.

[84] Organization for Economic Co-operation and Development (2013). The OECD indicators on Employment Protection Legislation. http://www.oecd.org/employment/emp/oecdindicatorsofemploymentprotection.htm.

[85] Work Health and Safety Act of Turkey Law No. 6331, Official Gazette Dated 30 June 2012 and No. 28339. https://www.mevzuat.gov.tr/mevzuat?MevzuatNo=6331&MevzuatTur=1&MevzuatTertip=5.

[86] European Commission Council Directive 89/391/EEC of 12 June 1989 on the Introduction of Measures to Encourage Improvements in the Safety and Health of Workers at Work. Retrieved from Access to European Union Law. http://data.europa.eu/eli/dir/1989/391/oj.

[87] Vocational Training Act of Turkey Law No. 3308. Official Gazette Dated 19 June 1986 and No. 19139. https://www.mevzuat.gov.tr/mevzuat?MevzuatNo=3308&MevzuatTur=1&MevzuatTertip=5.

[88] Başkan Takaoğlu Z.E., Celenk K., Olmezoğlu Iri N.I. (2018). The problems of occupational safety specialists. Gümüşhane Univ. J. Health Sci..

[89] Güllüoğlu E.N., Güllüoğlu A.N. (2019). Analysis of employment and work accidents in Turkish Construction Sector. Karaelmas J. Occup. Health Saf..

[90] Favi C., Germani M., Marconi M. (2017). A 4m approach for a comprehensive analysis and improvement of manual assembly lines. Procedia Manuf..

[91] Uemura T., Kani Y., Yamada T., Hamamoto K., Ilyina A.D., Miller S.D., Rodionov A.A., Ono H. (2021). Improving Kaizen success in CIS countries: A 4m–7w–5k checklist to elicit ideas from managers and workers. Glob. Bus. J..

[92] Gürcanlı G.E. (2008). The current situation in occupational safety in the world and in Turkey and the construction sector. Mühendislikte Mimar. Plan. Ölçü.

[93] Guthrie J.P. (2001). High-involvement work practices, turnover, and productivity: Evidence from New Zealand. Acad. Manag. J..

[94] Tanner J. (2017). The Psychology of Motivating Employees Through Training and Development. https://trainingindustry.com/blog/performance-management/the-psychology-of-motivating-employees-through-training-and-development/.

[95] Myers D. (2013). Construction Economics: A New Approach.

[96] Sezgin A.G.Ş., Aşarkaya A. (2016). İnşaat Sektörü [Construction Sector].

[97] Kart E. (2016). Subcontracted forms and “intermediary” agents of employment relationships: Foremen in the construction sector. Gaziantep Univ. J. Soc. Sci..

[98] Cockburn A. (2001). Writing Effective Use Cases.

[99] Republic of Turkey Ministry of Development (2019). Indicators of PPP Projects. http://www.sbb.gov.tr/wp-content/uploads/Yatirimlar/KOI%20Proje%20Gostergeleri.xlsx.

[100] Bilim A., Çelik O.N. (2018). General assessment of work accidents caused in the construction sector in Turkey. Omer Halisdemir Univ. J. Eng. Sci..

[101] Güvel Ş.T., Laptalı Oral E. (2018). Determining the application level of occupational health and safety legislation in the construction sector in Turkey. Çukurova Univ. J. Fac. Eng. Archit..

[102] Güvel Ş.T., Laptalı Oral E. (2021). Factors affecting personal protective equipment usage of construction workers. Cukurova Univ. J. Fac. Eng..

[103] Şengönül T. (2021). Investigation of Personal Protective Equipment Usage in the Construction Sector of Trabzon Province. Master’s Thesis.

[104] Biçkes B., Gürcanlı E. (2020). A Method for the use of health and safety management systems in construction project management. Int. J. Adv. Eng. Pure Sci..

[105] Akınbingöl A.G. (2016). Health and Safety Plan in Building Constructions. OHS Expertise Thesis.

[106] Korkmaz A.V. (2020). Evaluation of Large-Scale Construction Site for Occupational Health and Safety. TÜBAV Bilim.

[107] Yılmaz M. The Effect of the Principal Employer-Subcontractor Relationship on the OHS Process in the Building Sector. Önlem, 2016, August. https://www.sagedam.com/yapi-sektoru-asil-isveren-altisveren-iliskisi-4857-is-kanunu/.

[108] Akboğa K.Ö., Dikmen S.Ü., Gürcanlı G.E., Bayram İ., Baradan S. (2018). A field study about the development of occupational safety expertise system. J. Balikesir Univ. Inst. Sci. Technol..

[109] Acemoğlu D., Robinson J.A. (2014). Why Nations Fail: The Origins of Power Prosperity and Poverty.

[110] Rodrik D., Aysan A.F., Dumludağ D. (2015). Kaliteli Büyümeye Yönelik Kurumlar: Nelerdir ve Nasıl Kazanılır. Kalkınmada Yeni Yaklaşımlar.

[111] Kang S.-Y., Min S., Won D., Kang Y.-J., Kim S. (2021). Suggestion of an Improved Evaluation Method of Construction Companies’ Industrial Accident Prevention Activities in South Korea. Int. J. Environ. Res. Public Health.

[112] Gurmu A.T. (2019). Identifying and prioritizing safety practices affecting construction labor productivity: An empirical study. Int. J. Product. Perform. Manag..

[113] Woolley M., Goode N., Salmon P., Read G. (2020). Who is responsible for construction safety in Australia?. A STAMP analysis. Saf. Sci..

[114] Oswald D., Sherratt F., Smith S. (2018). Problems with safety observation reporting: A construction industry case study. Saf. Sci..

[115] Lozano-Díez R.V., López-Zaldívar O., Herrero del Cura S., Verdú-Vázquez A. (2019). Analysis of the impact of health and safety coordinator on construction site accidents: The case of Spain. J. Saf. Res..

[116] Ringen K., Dong X.S., Goldenhar L.M., Cain C.T. (2018). Construction safety and health in the USA: Lessons from a decade of turmoil. Ann. Work Expo. Health.

